# Analytical comparison between two hematological analyzer systems: Mindray BC‐5180 vs Sysmex XN‐1000

**DOI:** 10.1002/jcla.22955

**Published:** 2019-06-20

**Authors:** Jing Wang, Shengmei Zhao, Zhenguo Su, Xiangdong Liu

**Affiliations:** ^1^ Department of Laboratory Binzhou Medical University Yantai China; ^2^ Department of Clinical Laboratory Shandong Provincial Hospital Affiliated to Shandong University Jinan China

**Keywords:** Bland‐Altman, complete blood count, consistency, Mindray BC‐5180, Passing‐Bablok, Sysmex XN‐1000

## Abstract

**Background:**

To compare the Mindray BC‐5180 and Sysmex XN‐1000 instruments by analyzing the results of complete blood count in the external quality assessment in Shandong Province in 2018.

**Methods:**

In the external quality assessment, 10 batches of quality control materials were issued throughout the year. The test items were WBC, RBC, Hb, PLT, and HCT. The laboratories using Mindray BC‐5180 and Sysmex XN‐1000 were screened, and the results were analyzed by *t* test, Passing‐Bablok regression analysis, and Bland‐Altman analysis.

**Results:**

Thirty‐six laboratories using Mindray BC‐5180 instruments and thirty‐six laboratories using Sysmex XN‐1000 instruments were screened, and the average difference between the two instruments results is not significant (*P* > 0.05, *t* test). Passing‐Bablok regression analysis showed that the 95% confidence interval of the regression equation interception of each test item included 0, and the 95% confidence interval of the slope contained 1, *r* > 0.98, which showed that the correlation is good. The Bland‐Altman analysis showed that both instruments had more than 95% of the points within the 95% consistency limit (WBC97.2%, RBC95.6%, PLT97.2%, Hb96.7%, HCT97.5%). Within the consistency limit, the absolute value of the difference between the Mindray BC‐5180 instrument and the Sysmex XN‐1000 instrument is WBC 0.14%, RBC 0.26%, PLT 2.7%, and Hb 1.9%. HCT is 0.69%, and the difference is clinically acceptable.

**Conclusion:**

It can be considered that the two instruments have good correlation and consistency, and the two instruments can replace each other.

## 1 INTRODUCTION

Complete blood count is the most common project in clinical laboratories by detecting various blood cell components in human blood, assisting in clinical diagnosis and identification of blood diseases and other systemic diseases. With the continuous development of laboratory medical technology, a large number of advanced testing instruments and equipment have been widely used in the field of medical testing, greatly improving the efficiency of laboratory testing and the accuracy of testing.^1^, ^2^ At present, the hematological analyzer systems used in China are the Chinese Mindray series and the Japanese Sysmex series. Although the detection principle of the two instruments is basically the same, there are certain differences in different models, internal structures, reagents, calibrators, etc, which may lead to deviations in the test results and affect clinical analysis.

In 2018, the Shandong Provincial Clinical Testing Center organized the clinical laboratory of the whole province to carry out the external quality assessment of the complete blood count. A total of 10 batch quality control materials were issued throughout the year. In this study, the laboratories using Mindray BC‐5180 and Sysmex XN‐1000 were screened, and the results of external quality assessment were analyzed to compare the consistency and difference between the two instruments. Improve laboratory testing capabilities and ensure the accuracy and reliability of laboratory results.^3^, ^4^

## 2 MATERIALS AND METHODS

### 2.1 Specimens

In 2018, the Shandong Provincial Clinical Testing Center organized the clinical laboratory of the whole province to carry out the external quality assessment of the complete blood count. A total of 10 batch quality control materials were issued throughout the year. The quality control materials cover the high, medium, and low concentrations. After the laboratory receives the quality control materials, carefully check the integrity of the packaging and contents, and immediately store them at 2‐8°C after opening. The participating laboratories will simultaneously test with the laboratory routine specimens within the prescribed time. The test items are WBC, RBC, Hb, PLT, and HCT. The test results will be returned to the Provincial Clinical Testing Center within the specified time. The report includes the results of the test project, the batch number of the control material, the brand of the instrument, the place of origin, the model, the principle of measurement, and the manufacturer of the reagents and calibrators. The results of the laboratory using Mindray BC‐5180 and Sysmex XN‐1000 were selected. Significant outliers were then removed by the Grubbs test, and abnormal results caused by hemolysis, lipemia and control material degradation were also removed. The remaining data were used for the next step analysis.

### 2.2 Instruments

Mindray BC‐5180, manufacturer: Chinese Mindray company. Detection range: for blood cell count, white blood cell classification, hemoglobin concentration measurement in clinical examination. Detection principle: The analyzer uses the Coulter principle to detect the number and volume distribution of white blood cells, basophils, red blood cells, and platelets; the hemoglobin concentration is measured by colorimetry; the four‐category statistical count of white blood cells is obtained by semiconductor laser flow cytometry. On this basis, the analyzer calculates the remaining parameter results.

Sysmex XN‐1000, manufacturer: Japanese sysmex company. Detection range: for blood cell count, white blood cell classification, and hemoglobin concentration measurement in clinical examination. Detection principle: For white blood cells, optical detection of semiconductor laser and flow cytometry are used; for red blood cell and platelet counting, sheath flow direct current (DC) detection is used. For the hemoglobin content, the sodium lauryl sulfate hemoglobin assay was used for detection.

The reagents, controls and calibrators used in the two instruments are original and are operated according to the operating instructions provided by the instrument manufacturer.

### 2.3 Comparison of test results between the two instruments

The results of the laboratory using Mindray BC‐5180 and Sysmex XN‐1000 were selected. The two groups of test results that conformed to the normal distribution were tested by *t* test. According to the different test items, the difference between the two instruments was compared. The difference was statistically significant at *P* < 0.05.

### 2.4 Passing‐Bablok regression analysis

Passing‐Bablok regression method was used to analyze the results of the two instruments. The results of five items of WBC, RBC, Hb, PLT, and HCT were determined by Sysmex XN‐1000 (*y*), and the results of five items were determined by Mindray BC‐5180 (*x*) Perform a linear regression analysis to calculate the regression equation. The intercept is a measure of the systematic difference between the two instruments. If the 95% confidence interval of the intercept does not include 0, there are systematic errors in the two instruments. The slope is a measure of the difference in the ratio between the two instruments. The 95% confidence interval for the slope does not contain 1, and there is at least a proportional difference between the two methods.^5^ Passing‐Bablok regression analysis evaluates the correlation between the two instruments. Pearson's test was used to obtain the correlation coefficient. When the correlation coefficient *r* ≤ 0.35, the correlation degree is low; *r* is 0.36‐0.67, the correlation degree is moderate; and *r* is 0.68‐1.00, the correlation degree is high.^6^

### 2.5 Bland‐Altman deviation analysis

The test results of the two instruments in each laboratory were input into the MedCalc software for Bland‐Altman analysis, and the deviation map was drawn. The Bland‐Altman deviation map is a two‐dimensional Cartesian coordinate, where the *x*‐axis of the abscissa represents the average of the results of the two instruments, and the *y*‐axis of the ordinate represents the percentage of the difference between the two instruments and the average value of the sample. The upper and lower horizontal lines in the figure represent the upper and lower limits of the 95% consistency limit, expressed by mean +1.96 SD and mean −1.96 SD, where mean is the average and SD is the standard deviation. If the scatter is evenly distributed on the lower side of the *Y* = 0 horizontal line, most of the scatter is within the consistency limit, and the consistency limit is narrower within the clinically recognized boundary value, indicating that the two methods have higher consistency, and one method can replace the other method.^7^, ^8^ The difference was statistically significant at *P* < 0.05.

### 2.6 Statistical software

The *t* test was performed using SPSS 22.0 statistical software, and the Passing‐Bablok regression analysis and the Bland‐Altman deviation analysis were performed using Microsoft Excel 2010 and MedCalc Software. For each analysis, *P* value <0.05 was considered to be statistically significant.

## 3 RESULTS

### 3.1 Statistical analysis of instrument types used in laboratories in external quality assessment

In the 2018 external quality assessment of the complete blood count in Shandong Province, a total of 731 laboratories participated, mainly using four brand instruments: Mindray (210), Sysmex (305), NIHON KOHDEN (86), and HORIBA (51) and 79 other brand instruments. As can be seen from Table 1, the most commonly used instruments are Mindray BC‐5180 (36) and Sysmex XN‐1000 (36). The instruments of these two brands were selected for comparison analysis.

**Table 1:** Statistics of various brands of instruments used in the laboratories

Brand	Place of origin	Model	Number of laboratories
Mindray	China	BC‐5180	36
		BC‐5390	31
		BC‐6800	28
		BC‐5380	26
		BC‐6900	16
		BC‐5300	15
		BC‐3000	15
		BC‐2600	10
		BC‐5500	9
		BC‐5310	7
		BC‐5100	4
		BC‐1800	4
		BC‐5800	3
		BC‐300	3
		BC‐2800	3
Sysmex	Japan	XN‐1000	36
		XE‐2100	32
		XS‐1000i	32
		XS‐800i	30
		XT‐1800i	30
		XN‐2000	29
		KX‐21	21
		XN‐9000	20
		XT‐2000i	19
		Others	56
NIHON KOHDEN	Japan	MEK‐8222K	31
		MEK‐7222K	30
		MEK‐6318K	25
HORIBA	Japan	ABX‐Pentra	26
		ABX‐Micros	25
Others			79
Total			731

### 3.2 Comparison of test results between the two instruments

**Figure 1:**
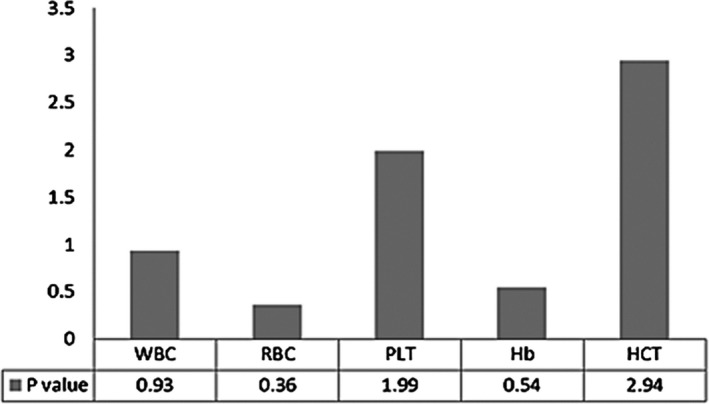
The results of two instruments, Mindray BC‐5180 and Sysmex XN‐1000, were tested by *t* test. The *P* values of the five test items of WBC, RBC, PLT, Hb, and HCT were all >0.05

The results of the laboratory using Mindray BC‐5180 and Sysmex XN‐1000 were screened out. The test items were WBC, RBC, PLT, Hb, and HCT. Thirty‐six laboratories used the Mindray BC‐5180 instrument to obtain 360 test results according to different test items: WBC (360), RBC (360), PLT (360), Hb (360), and HCT ( 360). Similarly, the Sysmex XN‐1000 has 360 test results per test items. According to the different test items, the test results of the two instruments were, respectively, tested by *t* test. The *P* value is shown in Figure 1. The *P* value of each test item is >0.05, and the conclusion that the test results of the two instruments are different cannot be obtained.

### 3.3 Passing‐Bablok regression analysis

**Figure 2:**
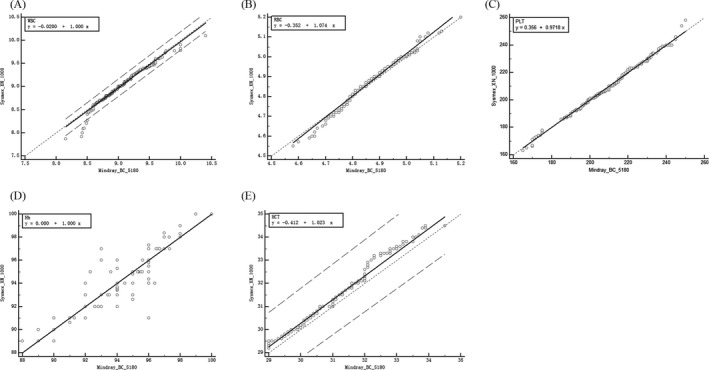
Comparison of test results of WBC, RBC, PLT, Hb, and HCT of Mindray BC‐5180 vs Sysmex XN‐1000 (A) Passing and Bablok regression analysis for WBC on 360 EQA results was *y* = −0.020 + 1.000*x*. (B) Passing and Bablok regression analysis for RBC on 360 EQA results was *y* = −0.352 + 1.074*x*. (C) Passing and Bablok regression analysis for PLT on 360 EQA results was *y* = 0.356 + 0.972*x*. (D) Passing and Bablok regression analysis for Hb on 360 EQA results was *y* = 0.000 + 1.000*x*. (E) Passing and Bablok regression analysis for HCT on 360 EQA results was *y* = −0.412 + 1.023*x*

The correlation analysis between Mindray BC‐5180 and Sysmex XN‐1000 was carried out by Passing‐Bablok regression analysis. The regression equations of each item are shown in Table 2, and the regression curve is shown in Figure 2. The results showed a linear correlation between the results of the two instruments. The 95% confidence interval for the intercept of the regression equation for each test item includes 0, and there is no systematic error between the two instruments. The 95% confidence interval of the slope contains 1, and there is no proportional difference between the two instruments, *r* is >0.98, and the correlation is good. From this result alone, the two detection methods can be substituted for each other.

**Table 2:** A passing–Bablok regression analysis for Mindray BC‐5180和Sysmex XN‐1000 comparison

	Equation	95% CI for intercept	95% CI for slope	*r*
WBC	*y* = −0.020 + 1.000*x*	−0.220 to 0.020	1.000 to 1.022	0.986
RBC	*y* = −0.352 + 1.074*x*	−0.434 to 0.010	1.000 to 1.091	0.997
PLT	*y* = 0.356 + 0.972*x*	0.000 to 0.796	0.963 to 1.006	0.989
Hb	*y* = 0.000 + 1.000*x*	0.000 to 0.000	1.000 to 1.000	0.993
HCT	*y* = −0.412 + 1.023*x*	−1.230 to 0.300	1.000 to 1.050	0.984

r: Pearson test was used for obtain the correlation coefficient.

### 3.4 Bland‐Altman deviation analysis

**Figure 3:**
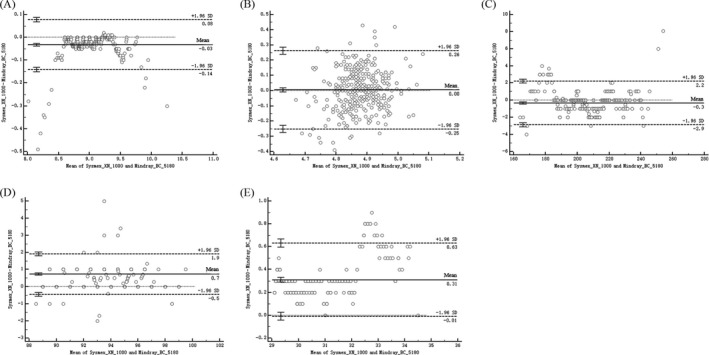
Bland‐Altman plots: comparing the WBC (A), RBC (B), PLT (C), Hb (D), and HCT (E) of Mindray BC‐5180 vs Sysmex XN‐1000. The dotted lines represent 95% limits of agreement

The test results of the two instruments in each laboratory were input into the MedCalc software for Bland‐Altman analysis, and the deviation map was drawn (Figure 3). Bland‐Altman analysis showed that both instruments had more than 95% of the points within the 95% consistency limit (WBC97.2%, RBC95.6%, PLT97.2%, Hb96.7%, HCT97.5%), meet the consistency requirements. Within the consistency limit, the absolute value of the difference between the Mindray BC‐5180 instrument and the Sysmex XN‐1000 instrument is WBC 0.14%, RBC 0.26%, PLT 2.7%, and Hb 1.9%. HCT is 0.69%, and the difference is clinically acceptable. Therefore, it can be considered that the results of the two instruments are consistent, and the two instruments are interchangeable.

## 4 DISCUSSION

Modern automated laboratory hematology analyzers allow the measurement of over 30 different hematological parameters useful in the diagnostic and clinical interpretation of patient symptoms.^9^, ^10^, ^11^ At present, the most commonly used instruments in the laboratory are Mindray BC‐5180 and Sysmex XN‐1000. Different detection systems use different methods and different anti‐interference ability, which will result in different results for different specimens on different detection systems. This difference may affect the clinician's development of a treatment plan. Therefore, when different instruments are used to detect the same item, the instrument needs to be compared to ensure the consistency of the test results.

In this study, the *t* test was performed on the test results of both instruments. The *P* value was >0.05. This result can only show that the average difference between the two instruments is not significant according to the current data, and it does not fully reflect the consistency between them. Moreover, the *P* value is affected by the degree of freedom, and the smaller the number of samples, the larger the *P* value.^12^ The *t* test is used to analyze the difference between the two test results, which can reflect the overall average error, but ignores the measurement difference between individuals. The *t* test is sensitive to systematic errors, but cannot take into account random errors. When the number of samples is sufficient, an insignificant difference can be tested. Passing‐Bablok regression analysis showed a linear correlation between the results of the two instruments. The 95% confidence interval for the intercept of the regression equation for each test item includes 0, and there is no systematic error between the two instruments. The 95% confidence interval of the slope contains 1, and there is no proportional difference between the two instruments, *r* is >0.98, and the correlation is good. From this result alone, the two detection methods can be substituted for each other.

When the systematic error (especially the proportional error) is large, the regression analysis can also show better consistency and is easy to produce the wrong conclusion. Therefore, regression correlation analysis cannot replace the consistency test. The *t* test can only be used to check whether the difference mean is close to 0. When the random error is large, the *t* test can show better consistency. Therefore, *t* test and regression analysis compare the two measurements are obvious one‐sidedness. So we introduce the Bland‐Altman method to compare the consistency of the two instruments. When the results are consistent, we can take into account the effects of random errors and systematic errors on the consistency results. It has unique advantages: the Bland‐Altman method is a graphical analysis method that makes the results of the analysis more intuitive. It allows us to combine multiple factors to judge the results, while taking into account the clinical acceptance of the maximum difference within the consistency limit. Extreme values can be clearly displayed.^13^ In this study, the Bland‐Altman analysis showed that both instruments had more than 95% of the points within the 95% consistency limit. Within the consistency limit, the absolute value of the difference between the Mindray BC‐5180 instrument and the Sysmex XN‐1000 instrument is clinically acceptable. It can be considered that the results of the two instruments are consistent, and the two instruments can be interchanged.

Our research uses *t* test, regression analysis, and Bland‐Altman method to combine quantitative analysis and qualitative analysis, and comprehensively consider the system error, random error, and measurement range limitation in the detection process, which can better reflect the difference between the two instruments.

In this study, the complete blood counts of Mindray BC‐5180 and Sysmex XN‐1000 instruments were compared, and the conclusions of the two types of instruments were consistent, which is the same as other people's research results.^14^, ^15^ In daily work, the influence of different inspection systems on the inspection results should be taken into consideration to avoid some medical errors. The operation should be strictly carried out in accordance with the standard operating procedure, and the comparison should be carried out regularly to ensure that the inspection results are accurate and consistent.
